# The influence of frailty syndrome on quality of life in elderly patients with type 2 diabetes

**DOI:** 10.1007/s11136-021-02829-x

**Published:** 2021-04-08

**Authors:** E. Bąk, A. Młynarska, C. Marcisz, R. Bobiński, D. Sternal, R. Młynarski

**Affiliations:** 1 Faculty of Health Sciences, University of Bielsko-Biala, ul. Willowa 2, 43-309, Bielsko-Biala, Poland; 2 Department of Gerontology and Geriatric Nursing, School of Health Sciences, Medical University of Silesia, Katowice, Poland; 3 Department of Electrocardiology and Heart Failure, School of Health Sciences, Medical University of Silesia, Katowice, Poland

**Keywords:** Frailty syndrome, Quality of life, Type 2 diabetes

## Abstract

**Introduction:**

There have been no comprehensive studies that assess the impact of frailty syndrome on quality of life (QoL) of patients with diagnosed type 2 diabetes. The purpose of the study was to assess the impact of frailty syndrome on QoL and depression symptoms of patients with type 2 diabetes.

**Methods:**

The study included 148 consecutive patients (aged ≥ 60y). The patients were divided into two groups according to the prevalence of the frailty syndrome: robust and frailty. For all of the patients that were included in the study, we used the Polish version of validated instruments: ADDQoL, TFI and BDI.

**Results:**

In the study group, 43.2% had been diagnosed with frailty syndrome. An analysis of QoL assessment depending on the prevalence of the frailty syndrome showed that patients who were robust (without recognized frailty syndrome) assessed QoL significantly better than patients with coexisting frailty syndrome. Robust patients did not have any severe depressive symptoms, whereas in the group of patients with the frailty syndrome 43.8% of the patients had a depression. 70.2% of the patients without any depressive symptoms were robust patients, meanwhile only 14% of the patients had frailty syndrome recognized.

**Conclusions:**

Frailty syndrome occurred in 43 percent of the patients with type 2 diabetes. This has a negative impact on QoL of patients. Depression is more common in patients with the frailty syndrome and diabetes.

## Introduction

In Poland, over three million people have diabetes and over one million are unaware of their illness. The prevalence of diabetes in people over 65 is estimated to be 25–30% [1]. In some populations, more than 30% of people over 65 years of age have diabetes, and more than half of all diabetic patients in the USA are more than 60 years old, they also have a reduced life expectancy of 7.3 to 9.5 years and a reduced chance of a good quality of life by 11.1 to 13.8 years [2–4]. What is more importantly, older people with diagnosed diabetes have a higher percentage of premature death and concomitant diseases such as hypertension, heart disease, cerebrovascular disease and stroke than those without diabetes and are also more likely to experience polypharmacy, depression, cognitive impairment, urinary incontinence, harmful falls and persistent pains, which are referred to as geriatric syndromes [5–7].

Frailty syndrome is a common and important geriatric syndrome characterized by a reduction in reserves and a resistance to stressors resulting from the accumulation of the decreased efficiency of various physiological systems, which in turn leads to a susceptibility to adverse consequences. The prevalence of frailty syndrome in the population of people with type 2 diabetes varies and it is reported to range from 5 to 48% based on various diagnostic criteria [8–12]. Frailty syndrome in patients with diagnosed diabetes can be an important risk factor for both mortality and disability. Several reports have suggested that an assessment of frailty must become a part of the routine assessment of older patients with diabetes [13–15].

Diabetes can affect the quality of life (QoL) of patients in many ways: emotionally, physically, financially and socially. Recent research suggests that diabetes often causes a number of psychological problems and mental disorders that do not cause pain but that affect the course of the disease and therapy [16, 17]. QoL in patients with diabetes is dependent on many sociodemographic and clinical factors. It has been shown that the reduction in QoL in diabetic patients is significantly affected by complications that are associated with this disease, i.e., the need to take insulin as well as the comorbidity of those complications [18–21].

There have been no comprehensive studies that assess the impact of frailty syndrome on QoL of patients with diagnosed type 2 diabetes. The purpose of the study was to assess the impact of frailty syndrome on quality of life and depression symptoms of patients with type 2 diabetes.

## Methods

The prospective study included 148 consecutive patients who were consulted in a diabetes outpatient clinic in the Regional Hospital in Bielsko-Biala (the Diabetic Clinic) and the Diabetic Unit of the Medi-Diab Non-Public Medical Center and the Diabetic Unit in Katowice between March 2016 and January 2017 who had been diagnosed with type 2 diabetes. All data were collected under the same condition, non-questionnaire data were collected under standardized condition using predefined methods and equipment. Additionally, data about treatment, diabetes compilation, comorbidity and anthropometric measurement were also collected during inclusion in the study visit. We collected following data: age, weight, height, BMI, waist circumference, WHR, Relative Fat Mass Index (RFM = 64—(20 × height/waist circumference) + (12 × gender) where: female = 1; male gender = 0), actually smoking, sociodemographic data, method of diabetes treatment, accidental glycaemia, fasting glucose, and diabetic complication – diabetic foot syndrome, diabetic nephropathy. Based on the population size, fraction size and maximum error at a 95% confidence level, the minimum number of patients in the sample was calculated – it was 144 patients needed to participate.

### Eligibility criteria

Patients that were included in the study had to meet the following inclusion criteria: they had been diagnosed with DM type 2 at least six months earlier, their consent to participate in the study and being more than 60 years of age. All patients were informed on the study protocol.

Excluded were patients with secondary diabetes, patients who had been diagnosed with acute inflammation that required treatment within the previous three months and patients taking immunosuppressive drugs, glucocorticoids, anti-inflammatory drugs, sedatives or psychoactive drugs, as well as patients who had been diagnosed with a malignant disease, thyroid and adrenal disorders and alcoholics. Patients who did not consent to participate in the study (*n* = 22) and patients who filled out questionnaires incompletely (*n* = 12) were excluded from the study.

Patients included in the study were divided into two groups depending on the prevalence of frailty syndrome:

- group 1 robust—without a recognized frailty syndrome,
- group 2 frailty—with a recognized frailty syndrome.

### Ethical considerations

The local ethics committee of the Bioethics Committee of the Beskidzka Regional Chamber of Physicians in Bielsko-Biala approved the study protocol (Consent No. 2016/02/11/1). The study protocol complied with the version of the Helsinki Convention that was current at the time the study was designed.

### Psychometric tools used in the research

For all of the patients that were included in the study, we used the Polish version of three validated instruments: The Audit of Diabetes-Dependent Quality of Life (ADDQoL), the Tilburg Frailty Indicator (TFI) and the Beck Depression Inventory (BDI).

The ADDQoL questionnaire is used to test QoL in patients with diabetes. It consists of two general questions about QoL: determining the measurement of the general current level of QoL, and the specific impact of diabetes on the quality of life. The next questions relate to 19 QoL domains without disease and the impact of diabetes on aspects of life. Each domain contains two components: impact (from -3, maximum negative impact of diabetes, up to + 1, positive effect of diabetes) and importance (3—very important, 0—not important at all). The result of the impact and importance assessment determines the value of the weighted impact (WI). The value of WI may vary from − 9 to + 3 for each of the tested ADDQoL domains. The lower the value of the weighted result, the worse the aspect of life in a given domain is assessed. The average value of the weighted effect (AWI) was calculated for the entire scale. The AWI result is the value obtained by dividing the sum of the weighted ratings by the number of relevant domains from -9 (maximum negative impact of diabetes) to + 3 (maximum positive impact of diabetes).

The ADDQOL scale that was adapted to Polish conditions by Bąk has a reliability of alpha = 0.93, which means that the scale is characterized by a good reliability index. The ADDQoL was applied in the studies with the consent and license received from the author, Clare Bradley (Health Psychology Research Unit, Royal Holloway, University of London via www.healthpsychologyresearch.com. The license for the Polish language version bore the number CB521) [22, 23].

The Tilburg Frailty Indicators is a simple diagnostic tool that takes into account a multidimensional approach to the state of frailty. It is based on the assessment of the physical, psychological and social indicators of functioning. The questionnaire consists of two parts. Part A (the determinants of frailty) contains questions related to the sociodemographic data as well as lifestyle, the occurrence of chronic diseases, traumatic events in the previous year and living arrangements. Part B, on the other hand, refers to the frailty component and contains questions about the three domains of frailty (physical domain, psychological domain, social domain). The questionnaire consists of three subscales: the physical subscale (0–8 points), which measures physical health, unintentional weight loss, difficulty walking, balance, hearing and vision problems, grip strength, and physical fatigue; the psychological subscale includes, among others, memory problems, depression, nervousness or anxiety, and the inability to cope with problems; The social subscale includes three elements: lonely life, lack social relations and lack of social support. eleven questions have two categories of answers: “yes” and “no”, four questions also have an answer category “sometimes”. After recoding, the result ranges are as follows: 0–15 (general frailty), 0–8 (physical frailty), 0–4 (mental frailty), and 0–3 (social frailty). The total score is within the range of 0–15 points with 5 being the cut-off point for frailty. The instrument was adaptation and translation for the Polish cultural context according to Uchmanowicz et al. internal Cronbach alpha coherence for this measurement it was 0.74 [24, 25].

The Beck Depression Inventory is a scale of self-report measure designed to measure severity of depression. It was published in 1961 and improved in 1971. It consists of 21 questions about a patient's mood in the previous seven days. Each question has four answers that are related to the increasing severity of symptoms. Each answer is assigned a score of 0–3 and the sum of the points indicates the severity of depression: 0–9 points – no depression, 10–18 points – mild depression, 19–29 points – moderately severe depression and > 30-point – severe depression. The total score is within the range of 0–63 points – higher total scores indicate more severe depressive symptoms [26, 27].

### Statistical analysis

The statistical analysis was performed using the Statistica 13 software. A *p*-value < 0.05 was considered significant. The normality of the distribution of variables was checked with the Shapiro–Wilk test. For any qualitative data and quantitative data that did not have a normal distribution, the non-parametric U Mann–Whitney tests were used and for the quantitative parameters with a normal distribution, the Student’s *t*-tests were used. The Spearman correlation coefficient r was used to correlate level of frailty and the severity of depressive symptoms. To calculate of Cohen’s d effect size was use following calculator (https://www.psychometrica.de/effect_size.html#transform), depending on the type of test. For non-parametric test eta square was calculated and next this parameter was transformed into Cohen’s d. This parameter show the strength of the relationship between variables and allow to determine the meaning of such a relationship.

## Results

### Characteristics of the study group

The group of patients without recognized frailty syndrome – robust – was younger, had a smaller body mass of 81.5 kg (71.5–93.5 kg) and a lower mid-waist and hip circumference. In this group, there were more people with a higher or secondary education as well as people who were still actively working. In the group of patients that had been diagnosed with frailty syndrome, there were more retirees and people who had smoked in the past – longer those smokers compared to the robust group. The mean time from diagnosis of diabetes to the inclusion visit was 12.86 ± 9.30 years. The sociodemographic characteristics are summarized in Table 1.

**Table 1:** Characteristics of the patients that were included in the study

	Frailty *n* = 64			Robust *n* = 84			*p*	d_Cohen_
	Median number	1st quartile %	3rd quartile	Median number	1st quartile %	3rd quartile		
Age [years]	68.5	65	75	66	60	69	0.002	0.521
Height [cm]	170	163	174	168	162	174.5	0.38^t^	–
Man	170	173	175.5	168.8	175	180	0.490^t^	–
Woman	158	163	170	160	164	168	0.769^t^	–
Weight [kg]	88.5	80	96	81.5	71.5	93.5	0.02	0.388
Man	87	93	100	85.3	94	101.3	0.848	–
Woman	72	82	87	68	74	83	0.124^t^	–
BMI [kg/m^2^]	31.2	27.7	33.2	29.2	26.0	31.2	0.008	0.447
Man	29.1	31.2	34.0	27.5	29.9	34.4	0.313	–
Woman	27.4	30.8	32.0	25.2	27.3	30.7	0.025	0.066
Waist circumference	101.5	94	110	95	90	102	0.01	0.410
Man	100	108	114	93.8	100	112.5	0.119	–
Woman	89	94	101	89.5	93	98.5	0.590^t^	–
Hip circumference	110	97	118	102	93.5	110	0.009	0.439
Man	97	112	120	97.3	106	115.3	0.074	–
Woman	95	105	113	90.0	100	110	0.173	–
WHR	0.9	0.9	1.0	0.9	0.9	1.0	0.52	–
Man	0.9	1.0	1.1	0.9	1.0	1.0	0.495^t^	–
Woman	0.8	0.9	0.9	0.8	0.9	1.0	0.126	–
RFM	34.0	31.6	36.6	35.1	29.4	37.5	0.41	–
Man	28.9	32.3	34.2	26.6	29.1	34.0	0.099	–
Woman	34.9	37.1	38.9	35.1	36.7	39.2	0.888	–
Gender							0.05	0.324
Female	27	42.2		49	58.3			
Male	37	57.8		35	41.7			
Place of living							0.21	–
Rural	48	75		55	65.5			
Urban	16	25		29	34.5			
Education							0.02	–
Primary	21	32.8		17	20.2			
Vocational	25	39.1		28	33.3			
Secondary	14	21.9		30	35.7			
Higher	4	6.3		9	10.7			
Marital status							0.22	–
Unmarried	5	7.8		5	5.9			
Married/living with partner	32	50		56	66.7			
Widow/widower	19	29.7		15	17.9			
Divorced	8	12.5		8	9.5			
Professional status							0.03	0.505
Working	11	17.2		31	36.9			
Unemployed	1	1.6		4	4.8			
Pensioner	4	6.3		4	4.8			
Retired	48	75		45	53.6			
Type of work							0.25	–
Mental	16	28.1		26	37.7			
Physical	41	71.9		43	62.3			
Smoking	28	43.8		27	32.1		0.15	–
Number of days	12.5	10	15.5	14	10	20	0.37	–
Number years smoking	30	20	40	20	12	26	< 0.001^t^	0.517
Treatment								
Insulin	47	73.4		42	50		0.01	0.493
Oral medications	5	7.8		10	11.9			
Dietary	12	18.8		32	38.1			

^t^ Student’s test

*BMI* Body Mass Index, *RFM* Relative Fat Mass Index, *WHR* Waist-Hip Ratio

In the study group, 43.2% had diagnosed frailty syndrome, the average value of the points that were obtained using TFI questionnaire was 4.17 ± 2.98 points. According to the domains division, the psychological domain got 1.16 ± 1.29 points out of four (29%), the physical domain got 2.24 ± 1.61 points out of eight (28%) and finally the social domain, 0.76 ± 0.75 out of three (25%).

An analysis of QoL assessment depending on the prevalence of the frailty syndrome showed that patients who were robust (without recognized frailty syndrome) assessed QoL significantly better than patients with coexisting frailty syndrome. An analysis of the occurrence of depressive disorders showed statistically significant differences in the study groups. Robust patients did not have any severe depressive symptoms, whereas in the group of patients with the frailty syndrome as many as 43.75% of the patients had such disorders. Patients without any depressive symptoms, 70.24% of the robust patients, were compared to only 14.04% of the patients with frailty syndrome. All statistical differences should be treated as large clinically relevant. Detailed data are presented in Table 2.

**Table 2:** Assessment of the quality of life, the frequency of depression depending on the occurrence of frailty syndrome

Parameter	Frailty, *n* = 64	Robust, *n* = 84	All	*p*	d_Cohen_
ADDQoL	− 2.84 (-4.68;-1.87)	− 1.34 (-2.24;-0.70)	− 1.97 (-3.43;-1.03)	< 0.001	1.101
BDI	22.5 (15;33.5)	7.5 (4;12.5)	12.5 (7;21.5)	< 0.001	1.888
Without depression	9 (14.06%)	59 (70.24%)	68 (45.94%)	< 0.001	1.916
Mild depression	16 (25%)	23 (27.38%)	39 (26.35%)		
Moderate depression	11 (17.19%)	2 (2.38%)	13 (8.78%)		
Severe depression	28 (43.75%)	0 (0%)	28 (18.92%)		

Data presented as medians (I quartiles; III quartiles)

*ADDQoL* Audit of Diabetes-Dependent Quality of Life, *BDI* Beck Depression Inventory

The correlations between the level of frailty and the severity of depressive symptoms showed that the greater severity of frailty and its components, the greater severity of the depressive symptoms: BDI versus global frailty: *r* = 0.7841, *p* < 0.05, BDI versus the physical components: *r* = 0.5503, *p* < 0.05, BDI versus the psychological components: *r* = 0.9184, *p* < 0.05 and BDI versus the social components: *r* = 0.3517, *p* < 0.05.

Robust patients had a better quality of life score in domains 1 to 17 compared to patients with frailty syndrome. The higher the score, the better QoL. There were no significant differences between the groups in the freedom to eat and freedom to drink domains. The overall quality of life was better in patients without frailty syndrome *p* < 0.001. Patients with frailty syndrome had the lowest quality of life scores in the family life, people's reaction, friendship and social life domains, while robust patients had the worst quality of life scores in the domains of people’s reaction, living conditions and working life. All statistical differences should be treated as intermediate clinically relevant. Detailed data are presented in Table 3.

**Table 3:** Distribution of the ADDQoL responses by the weighted impact score for both the robust and frail groups

Weighted impact								
Domain	All	Rank	Frailty	Rank	Robust	Rank	*p*	d_Cohen_
1 Leisure activities	−2 (−4;−1)	14	−4 (−6;−2)	15	−2 (−4;0)	16	< 0.001	0.71
2 Working life	−1 (−3.5;0)	4	−4 (−6;0)	13	0 (−2;0)	3	0.02	0.371
3 Journeys	−1 (−3;0)	7	−2 (−4;0)	5	0 (−1.5;0)	9	< 0.001	0.588
4 Holidays	−2 (−4;0)	12	−3 (−4;−1)	8	−1 (−3;0)	12	< 0.001	0.605
5 Physical health	−2 (−4;−1)	16	−4 (−6;−2)	18	−1 (−3;0)	13	< 0.001	1.085
6 Family life	−2 (−3;0)	11	−3 (−4;−2)	10	0 (−2;0)	6	< 0.001	0.854
7 Friendship & social life	0 (−2;0)	3	−1 (−4;0)	3	0 (−1.5;0)	4	0.001	0.55
8 Personal relationship	0 (−4;0)	9	−3 (−6;0)	9	0 (−2;0)	8	< 0.001	0.581
9 Sex life	−2 (−3;0)	6	−2 (−4;−2)	7	−1 (−2;0)	10	< 0.001	0.68
10 Physical appearance	−1 (−2;0)	8	−2 (−4;0)	4	−1 (−2;0)	11	0.03	0.355
11 Self-confidence	−1 (−3;0)	5	−2 (−4;0)	6	0 (−2;0)	7	0.002	0.494
12 Motivation	−2 (−4;0)	17	−4 (−6;−2)	17	−2 (−3;0)	14	< 0.001	1.052
13 People’s reaction	0 (−2;0)	1	−0.5 (−4;0)	2	0 (0;0)	1	< 0.001	0.51
14 Feelings about the future	−4 (−9;0)	19	−6 (−9;−4)	19	−2 (−4;−1.5)	18	< 0.001	0.856
15 Financial situation	−2 (−4;0)	13	−4 (−6;−2)	14	−2 (−3;0)	15	< 0.001	0.598
16 Living conditions	0 (−2;0)	2	−1 (−2;0)	1	0 (−1;0)	2	0.002	0.473
17 Dependence on others	0 (−4;0)	10	−3 (−6;0)	11	0 (−2;0)	5	< 0.001	0.096
18 Freedom to eat	− 3 (−6;−1)	18	−4 (−6;−2)	16	−2 (−6;−1)	19	0.10	–
19 Freedom to drink	− 2 (−4;0)	15	−2.5 (−6;0)	12	−2 (−4;0)	17	0.11	–

## Discussion

Worldwide, the number of aging individuals is increasing and the incidence of diabetes is also rapidly increasing. Therefore, the number of elderly people who have been diagnosed with diabetes has also increased and the combination of aging and diabetes contributes to functional disability. A cardiovascular health study showed that 25% of patients with frailty syndrome had diabetes and that more than 18% had prefrail syndrome. Only 12% of patients without frailty syndrome were diagnosed with diabetes [8–12]. The prevalence of frailty syndrome in the population of people with type 2 diabetes varies and, depending on the authors and the diagnostic criteria that are adopted, ranges from 5 to 48% [8]. In the population in this study, frailty syndrome occurred in 43.2% of the patients with diagnosed diabetes. Frailty syndrome is considered to be an important risk factor for both mortality and disability in older patients with type 2 diabetes [13].

In the studies of Ottenbacher, Hubbard and Cacciatore, it was demonstrated that elderly patients with diabetes were more likely to present frailty syndrome than their non-diabetic peers. These studies also provided data on the prognosis in patients with frailty syndrome and diabetes. The occurrence of frailty syndrome in patients with diabetes was an independent risk factor for death, disability and cognitive impairment and was also associated with a decrease in QoL [28–30]. The ESTHER study in Germany (Epidemiologische Studie zu Chancen der Verhütung, Früherkennung und optimierten Therapie chronischer Erkrankungen in der älteren Bevölkerung) and the Whitehall II study showed that the incidence of frailty syndrome was three- to fivefold higher in patients older than 65 years who had been diagnosed with diabetes compared to the general population [31, 32].

Regarding QoL domains in the conducted study, which concerned a specific group of patients – only patients over 60 with diagnosed type 2 diabetes, many aspects of functioning and quality of life were affected by diabetes. Although diabetes negatively affected all aspects of QoL, this effect was more significant in patients with frailty syndrome. While the use of non-pharmacological treatment positively influenced the assessment of QoL, the occurrence of complications including neuropathy, diabetic foot syndrome caused a decrease in QoL. The occurrence of frailty syndrome had a negative impact on QoL of the studied population regardless of the domain.

In our study, the number of medications (oral medication and insulin) being taken by patients in the frail group was greater than for those in the non-frail group. Literature shows that diabetic patients often use many medications that are required for tight glycemic control and often have comorbidities that also require many medications. Polypharmacy is associated with the more frequent occurrence of frailty syndrome in older people. While co-morbidities are often observed in older populations, two studies that used statistical analyses showed that the polypharmacy that controlled many potential confounders including co-morbidities was associated with frailty [33, 34].

When investigating patients with diabetes using the SF-20 questionnaire, Glasgow et al. showed that factors such as a low level of education, older age, female gender, type of insurance, social status, the number of complications of diabetes, the number of comorbidities and a low level of physical activity during everyday activities caused a worse quality of life. These results were also confirmed in this study; the factors lowering the quality of life were more pronounced in the group of patients with the frailty syndrome. The occurrence of frailty syndrome is probably a factor that worsens QoL of patients with type 2 diabetes [34, 35].

In population studies that were conducted in the group of 3010 people in South Australia, depression was diagnosed in 24% of the patients with diagnosed diabetes. QoL in the domains of physical and mental functioning was statistically significantly lower among patients with diabetes and depression compared to the respondents with non-depressive diabetes [19]. It is believed that at any given time about 33% of people with diabetes have symptoms of depression that require treatment [36, 37]. In the our study, depression was diagnosed in 54.06% of the patients, and was found more often in patients with diabetes and diagnosed frailty syndrome compared to the robust patients (without frailty) with diabetes.

Quality of life is the ultimate goal of all health interventions. Quality of life measures physical and social functional and perceived physical and mental well-being. People with the diabetes have a poorer quality of life than people without chronic disease, but also better quality of life than people with most other serious chronic diseases. Numerous demographic and psychosocial factors influence the quality of life [38]. In our opinion, frailty syndrome may be one of the syndromes that worsen the quality of life in diabetes. Diabetes influence the quality of life through macrovascular complications and associated extravascular comorbidities. Future research areas should include transcultural and ethnic aspects and the effects of lifestyle interventions [39].

Numerous studies suggest that the assessment of frailty syndrome should become part of the routine assessment of elderly patients with diabetes [14, 15]. In our opinion, this statement will also be important in the population of elderly people diagnosed with diabetes. Such a procedure may allow for earlier detection of patients at risk of depression symptoms and deterioration of the quality of life.

## Conclusion

Frailty syndrome occurred in 43 percent of the patients with type 2 diabetes. This has a negative impact on quality of life of patients. Depression is more common in patients with the frailty syndrome and diabetes.

## Study limitations

The limitation of this trial may be the use of the only one frailty syndrome identification tool. There are no guidelines that facilitate the choice of a specific tool in the specific disease. Another limitation is relatively low number of patients enrolled, but within the estimated minimum sample. Finally, non-randomized nature of the study is also limitation.

## Data Availability

The datasets used and analyzed during the current study are available from the corresponding author on reasonable request.

## Abbreviations

ADDQoL: Audit of Diabetes-Dependent Quality of Life
AWI: Average of Weighted Impact
BDI: Beck Depression Inventory
BMI: Body mass index
p: Predictor significance level
QoL: Quality of Life
RFM: Relative Fat Mass Index
TFI: Tilburg Frailty Indicator
WHR: Waist-Hip Ratio
WI: Weighted Impact
